# Image Processing in Ophthalmology

**DOI:** 10.1155/2018/1545632

**Published:** 2018-05-20

**Authors:** Robert Koprowski, Anna Nowińska, Sławomir Wilczynski, Michele Lanza, Vasyl Molebny, Renato Ambrosio

**Affiliations:** ^1^Department of Biomedical Computer Systems, Faculty of Computer Science and Materials Science (Institute of Computer Science), University of Silesia, Ul. Będzińska 39, 41-200 Sosnowiec, Poland; ^2^Chair and Department of Ophthalmology, School of Medicine with the Division of Dentistry in Zabrze, Medical University of Silesia, Katowice, Poland; ^3^Department of Basic Biomedical Science, School of Pharmacy with the Division of Laboratory Medicine in Sosnowiec, Medical University of Silesia, Sosnowiec, Poland; ^4^Multidisciplinary Department of Medical, Surgical and Dental Sciences, Università della Campania Luigi Vanvitelli, Napoli, Italy; ^5^Academy of Technological Sciences of Ukraine, National University of Kiev, Kiev, Ukraine; ^6^2VisareRIO, Instituto de Olhos Renato Ambrósio, Rio de Janeiro, RJ, Brazil; ^7^Rio de Janeiro Corneal Tomography and Biomechanics Study Group, Rio de Janeiro, RJ, Brazil; ^8^Brazilian Study Group of Artificial Intelligence and Corneal Analysis-BRAIN, Rio de Janeiro, RJ, Brazil; ^9^Department of Ophthalmology/DECIGE, Federal University of the State of Rio de Janeiro, Rio de Janeiro, RJ, Brazil; ^10^Department of Ophthalmology, Federal University of São Paulo, São Paulo, SP, Brazil

This special edition concerns image processing in ophthalmology. This issue is related to a variety of modern imaging methods. Precise discernment of a disease, its correct diagnosing, effectual planning, and successful realization of treatment, especially surgical, in any branch of medicine, including ophthalmology, cannot be provided today without specific techniques of image processing and quantitative data. Each and every obtained quantitative result is the outcome of image analysis and processing algorithms implemented in a software. However, the software is not always perfect. Even today, the software does not provide fully automatic, reproducible, and quantitative results in the case of high interindividual variability. Therefore, the software in ophthalmology implementing corresponding algorithms must be tailored for specific applications—analysis of the retina, cornea, and so on. This area of image processing has been addressed in six articles included in this special edition.

Imaging the blood vessels, their accentuation, or even extraction is still the area of interest. It concerns the selection of image analysis and processing methods to extract blood vessels. It is a real art to find a segmentation method whose results are independent of the image angle (rotation). Z. Yavuz and C. Köse proposed a method using four stages: preparation of a dataset for segmentation; Gabor, Frangi, and Gauss filtration and top-hat transform; and K-means and fuzzy c-means clustering and removal of falsely segmented isolated regions. The obtained results relate to the K-means clustering method—95.94% and 95.71% of accuracy for STARE and DRIVE databases. A similar issue was discussed in the article written by R. Ramli et al., who used a difference of Gaussian pyramid with a saddle detector. This pyramid was tested on the Fundus Image Registration Dataset consisting of 134 retinal image pairs. The authors' results showed that D-Saddle registered 43% of retinal image pairs with an average registration accuracy of 2.329 pixels. The method proposed by R. Ramli et al. provides much better results compared to the results known today. A new method of analysis and interesting results are also presented in the work of A. Lyssek-Boroń et al. In this work, the authors analyse retinal OCT. By comparing the group with open-angle glaucoma and the group without glaucoma, they demonstrated that the retinal nerve fibre layer thickness in the temporalis quadrant decreases significantly 6 months after surgery. Measurements of corneal thickness in the eyes with pseudoexfoliation syndrome are presented by K. Krysik et al. They showed that the mean CCT in the eyes with PEX syndrome measured with three instruments was thicker compared to that in the normal eyes. The results concerned 128 eyes of patients with diagnosed PEX and 112 normal eyes.

Two of the articles concern the cornea and pupil. One of the papers written by P. Kasprowski and K. Harezlak is devoted to the vision diagnostics and treatment system for children with disabilities. This system enables to track eyeball motility. The presented system is equipped with specialized software. This system allows stimulation of children's vision with a dedicated stimulus and post hoc analyses of recorded sessions. It is therefore an extremely useful tool for both an ophthalmologist and neurologist. The other article written by R. Koprowski and S. Wilczyński focuses on the dynamics of the cornea. Monitoring of the cornea during intraocular pressure measurements provides additional parameters such as amplitude of vibrations or their phase. This is discussed in detail in the mentioned article.

In summary, this special edition is mainly devoted to the methods based on the detection and segmentation of vessels and arterioles of the retina; there are also articles discussing OCT, eye tracking, and a high-speed Scheimpflug camera. We hope that this special edition will bring new knowledge and will be interesting and inspiring for the readers.

*Robert Koprowski* *Anna Nowińska* *Sławomir Wilczynski* *Michele Lanza* *Vasyl Molebny* *Renato Ambrosio*

